# Ultra-Transparent and Multifunctional IZVO Mesh Electrodes for Next-Generation Flexible Optoelectronics

**DOI:** 10.1007/s40820-024-01525-y

**Published:** 2024-09-26

**Authors:** Kiran A. Nirmal, Tukaram D. Dongale, Atul C. Khot, Chenjie Yao, Nahyun Kim, Tae Geun Kim

**Affiliations:** 1 School of Electrical Engineering, Korea University, Anam-ro 145, Seongbuk-gu, Seoul, Republic of Korea; 2 Computational Electronics and Nanoscience Research Laboratory, School of Nanoscience and Biotechnology, Shivaji University, Kolhapur, 416004 India

**Keywords:** Self-cracking template, Vanadium-doped indium zinc oxide mesh, Organic solar cells, Organic light-emitting diodes, Flexible transparent memory

## Abstract

Mechanically durable transparent electrodes are essential for achieving long-term stability in flexible optoelectronic devices. Furthermore, they are crucial for applications in the fields of energy, display, healthcare, and soft robotics. Conducting meshes represent a promising alternative to traditional, brittle, metal oxide conductors due to their high electrical conductivity, optical transparency, and enhanced mechanical flexibility. In this paper, we present a simple method for fabricating an ultra-transparent conducting metal oxide mesh electrode using self-cracking-assisted templates. Using this method, we produced an electrode with ultra-transparency (97.39%), high conductance (*R*_s_ = 21.24 Ω sq^−1^), elevated work function (5.16 eV), and good mechanical stability. We also evaluated the effectiveness of the fabricated electrodes by integrating them into organic photovoltaics, organic light-emitting diodes, and flexible transparent memristor devices for neuromorphic computing, resulting in exceptional device performance. In addition, the unique porous structure of the vanadium-doped indium zinc oxide mesh electrodes provided excellent flexibility, rendering them a promising option for application in flexible optoelectronics.

**Supplementary Information:**

The online version contains supplementary material available at 10.1007/s40820-024-01525-y.

## Highlights

- Ultra-transparent vanadium-doped indium zinc oxide mesh (mIZVO) electrodes are fabricated using a self-cracking template.
- Fabricated electrodes are employed to realize flexible organic solar cell (OSC), organic light-emitting diode (OLED), and memristor devices. OSC exhibits 14.38% power conversion efficiency and OLED achieves 18.06% external quantum efficiency with mIZVO electrode.
- Flexible-transparent memristor based on mIZVO mimics various synaptic functions.

**Supplementary Information:**

The online version contains supplementary material available at 10.1007/s40820-024-01525-y.

## Graphical abstract

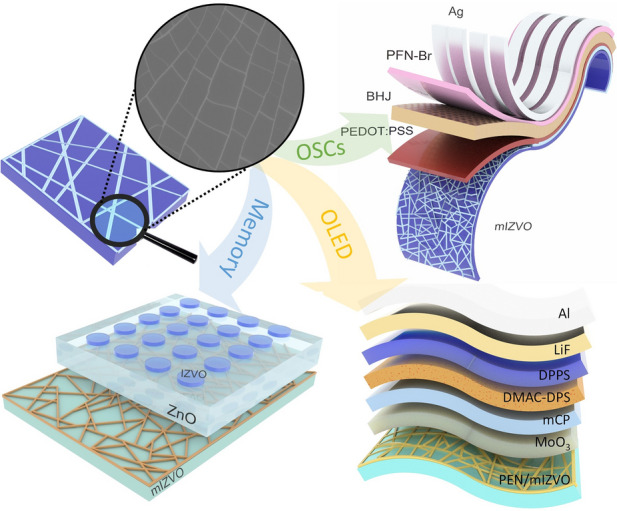

## Introduction

Considerable attention has been devoted to the domain of flexible and wearable electronic devices due to their light weight, versatile geometry, and their ability to be seamlessly integrated with next-generation technologies. As exemplified by smart clothing [1], electronic skin [2], and transparent heaters [3], cutting-edge applications are rapidly emerging in this futuristic electronic domain. Flexible transparent conductive electrodes (FTCEs) are indispensable elements when designing and developing flexible electronic devices and systems because they play a vital role in achieving bendability and stretchability [4]. To date, transparent conductive oxides (TCOs), notably indium tin oxide (ITO), are the most commonly used electrode materials for optoelectronic devices. ITO films are typically deposited onto transparent plastic substrates, such as polyethylene terephthalate (PET) or polyethylene naphthalate (PEN) [5]. However, the inherent rigidity and brittleness, high processing temperatures for crystallization, and significant light reflection of crystalline ITO impose limitations on its use in flexible optoelectronic devices [6, 7]. In comparison, amorphous ITO exhibits superior flexibility due to its non-crystalline structure, although it is inferior in terms of conductivity and transparency when compared to its crystalline form [8].

Currently, alternative materials are being researched to overcome the challenges linked with ITO in optoelectronic devices and ensure the establishment of a sustainable industry at the terawatt scale. To solve these challenges and maintain flexibility without compromising optical and electrical performance, various flexible transparent electrode materials have been developed recently, including conducting polymers [9, 10], conductive meshes [11–13], metal nanowires [14, 15], ultrathin metal films [16], carbon [17, 18], and two-dimensional materials [19, 20]. However, although there has been significant progress in advancing the practical applications of these materials, cost-effectiveness remains a major challenge. This is mainly attributed to the complexities associated with the fabrication methods and the intricate post-treatment and etching transfer processes, which are hindering widespread adoption [5]. In addition, these flexible transparent electrodes present another challenge in terms of the trade-off between sheet resistance and transmittance. Solution processes offer significant advantages for FTCEs, including mild processing conditions, high throughput, and the ability to micropattern diverse conductive nanomaterials into networks. Recent advancements, such as those introduced by Kim et al., have demonstrated the integration of multiple metal-oxide systems into nanocomposites for improved transparent electrodes. Despite these advances, integrating different materials into a single nanocomposite can be challenging. For example, issues such as phase separation and suboptimal interfacial bonding between the components can compromise the performance of the electrodes, affecting their electrical conductivity and mechanical stability [21]. To solve these problems, the one-step magnetron sputtering technique can simplify the fabrication process of amorphous TCOs (a-TCOs) on both rigid glass and flexible substrates, resulting in cost reductions when producing flexible optoelectronic devices compared to other complex methods. Moreover, the highly amorphous nature of a-TCOs means they are mechanically more flexible than ITOs.

Indium zinc oxide (IZO) thin films in the amorphous state have a uniform and isotropic microstructure that enhances flexibility and resistance to fractures due to the absence of grain boundaries. The literature suggests that introducing third cations with a higher ionic valence and a stronger metal-oxide bond dissociation energy than In^3+^ can effectively inhibit the creation of oxygen vacancies in In_2_O_3_ [5]. The carrier concentration of an IZO thin film is intricately related to both its transmittance and resistivity. Moreover, research has demonstrated that modifying the oxygen vacancies induces alterations in the optical and electrical characteristics of these thin films [22, 23]. Ko et al. developed a vanadium oxide-graded indium zinc oxide electrode that exhibited a promising Figure of Merit (FoM). However, practical application yielded only a modest power conversion efficiency (PCE) of 2.75% for organic solar cells (OSCs) [24]. More recently, Lan et al. highlighted the potential of indium-zinc-vanadium as an FTCE [5]. However, despite this advancement, electrode transparency remains an area that requires further improvement. Among the FTCEs, mesh electrodes are a promising alternative to ITO because they can effectively balance electric conductivity and optical transparency. In addition, they display enhanced mechanical flexibility due to their two-dimensional networks of an ultrathin conductive mesh. The high transparency of mesh electrodes also facilitates increased light transmission, particularly in near-infrared (NIR)-oriented optoelectronic applications [25, 26]. Various mesh electrode configurations have been developed that exhibit a stable sheet resistance (*R*_S_) [27, 28]. The mesh morphology can be obtained by hard template technologies (such as microsphere templates and laser ablation technology) and soft template technologies (including nanoimprinting, laser printing, and microchannel wetting techniques) [29]. Mesh structures that resemble cracks are common designs that can effectively balance the optical, electrical, and mechanical properties [30]. However, although mesh electrodes created via photolithography are effective, they are cost-intensive. To date, numerous metallic nanonetworks have been reported that offer high electrical conductivity and good mechanical strength [31–34]. For example, Han et al. reported the use of bioinspired networks for solar cells and flexible displays using leaf venations and spider web networks. Although these leaf mesh and spider web network-assisted fabrications offer unique and eco-friendly approaches, they face significant challenges in terms of uniformity, processing complexity, material properties, and environmental dependency [35]. Therefore, to overcome these limitations, there is a need to develop conductive metal oxide mesh electrodes with ultrahigh transparency, high conductivity, and robust mechanical durability. Recently, self-cracking template-assisted mesh electrode fabrication has become popular due to its cost-effectiveness [29]. Cracking templates can be designed using a variety of techniques, including grain boundary lithography [36], mud crack patterning [37], egg white [33], acrylic emulsion [38], and solvent-assisted cracking [31]. Among these, egg white templates offer a combination of biocompatibility, a controlled structure, cost-effectiveness, easy fabrication, and environmental sustainability, rendering them advantageous compared to other cracking templates. Peng et al. [39] reported on the fabrication of a metal ribbon network using an Ag seed layer deposited by thermal evaporation followed by Cu deposition through electroplating. Voronin et al. [40] fabricated copper mesh by employing an egg white-cracked template for transparent EMI shielding films. In these studies, egg white was used to fabricate metal meshes with relatively low transparency. Moreover, the high chemical reactivity, high cost, poor mechanical flexibility, and restricted process compatibility of metal meshes limit their use in different applications.

In response to the previous discussion, we introduce a vanadium-doped IZO mesh (mIZVO) electrode with ultrahigh transparency, high conductivity, and robust mechanical durability. The use of transparent conducting oxides helps to achieve ultra-transparency. This electrode is fabricated using a simple, self-cracking, template-assisted magnetron sputtering technique. The optical and electrical characteristics of the mIZVO electrode are selectively regulated through V doping by employing the co-sputtering technique. This method allows independent tuning of the work function (WF) and conductivity while preserving optical transmittance. We evaluated the effectiveness of the fabricated electrodes by integrating them into OSCs, organic light-emitting diodes (OLEDs), and flexible transparent (FT) memristor devices for neuromorphic computing. The developed electrode exhibits exceptional performance compared to those in the referenced articles. Finally, the device properties, based on the proposed electrodes, are evaluated and compared to those of IZO and ITO-based control devices. Our work on developing a flexible mIZVO electrode with multiple applications represents significant advancements in terms of material science and device engineering. Moreover, the versatility, performance, and durability of our electrode material provides new possibilities for flexible electronics. The developed flexible mIZVO electrode can be integrated into different applications, such as in-sensor, in-power, and in-memory computing systems, where flexible, efficient, and intelligent devices are ubiquitous and are transforming various fields, from consumer electronics to healthcare. In summary, this work presents the simplest protocol for fabricating ultra-transparent electrodes using mIZVO with excellent performance, offering a distinct and innovative approach compared to previous studies.

## Experimental Section/Methods

### Fabrication of Mesh Template Using Sacrificial Layer

PEN substrates with a thickness of 200 µm were purchased from AMG Korea. The substrates underwent a thorough ultrasonic cleaning process in isopropyl alcohol (IPA) and distilled water, with each treatment lasting for 10 min. This was followed by meticulous drying using nitrogen gas. The PEN substrates were then exposed to oxygen plasma for 3 min at 40 W. To prepare the sacrificial layer, an egg white was separated from the egg yolk and centrifuged at 3000 rpm. A yolk concentration of 0.1 g L^−1^ was then added to the fluid mobile fraction to form the sacrificial layer material, which was then coated on the plasma-treated PEN substrates using a glass rod. The layers spontaneously cracked under ambient conditions.

### Fabrication of Electrode

ITO, IZO, and V-doped IZO (IZVO) films with thicknesses of 150 nm were deposited onto the PEN substrates using an RF magnetron sputtering system (KVS-2000L, Korea Vacuum Tech). Details of the deposition conditions are listed in Table S1. To create the conducting network of IZVO (mIZVO), the self-cracking template was co-sputtered at a deposition rate of 0.19 nm s^−1^ using a magnetron sputtering power of 8 W. The mIZVO electrode featured a mesh structure and was fabricated by dissolving the egg white with distilled water.

### OSC Fabrication

All the electrodes deposited on the PEN substrates underwent UV/Ozone treatment. The PEDOT:PSS solution was spin-coated at 4000 rpm for 30 s and then annealed for 10 min at 120 °C on a hot plate, resulting in a thickness of 40 nm. After coating, the PEDOT:PSS-coated substrates were carefully transferred to a nitrogen-filled glove box. The ternary active layer composed of PM6:Y6:PC_71_BM (1:1:0.2 wt%, 16 mg mL^−1^) was spin-coated at 3500 rpm for 40 s, yielding a 120-nm-thick absorption layer. Subsequently, a 0.5 mg mL^−1^ PFN-Br layer was coated to achieve a thickness of 5 nm. Finally, the Ag electrode was deposited in a vacuum chamber. All the thermal depositions were executed under vacuum conditions with a pressure of less than 4 × 10^−6^ Torr.

### OLED Device Fabrication

Blue TADF OLEDs were crafted using 150-nm-thick ITO electrodes in a high vacuum chamber within a thermal evaporation system (Daedong High Tech, Republic of Korea) at a base pressure of 2.0 × 10^−7^ Torr. The reference device was assembled on a 150-nm ITO electrode with a 2-nm MoO_3_ layer as the HIL. The layered components were as follows: 40-nm-thick N, N‐ dicarbazolyl‐ 3,5‐ benzene (mCP) as the HTL, 28-nm-thick bis[4‐ (9,9‐ dimethyl‐ 9,10‐ dihydroacridine)phenyl] sulfone (DMAC-DPS) as the emissive layer (EML), 75-nm-thick diphenyl-bis(4-(pyridine-3-yl) phenyl)silane (DPPS) as the ETL, an 0.8-nm-thick LiF layer serving as the electron injection layer, and a 150-nm-thick Al layer as the cathode. Each layer was meticulously deposited and patterned using sequential shadow masks.

### Fabrication of Transparent Memory Device

When constructing the transparent flexible memory device, the mIZVO electrode served as the bottom electrode. A 40-nm-thick ZnO layer was deposited through sputtering, maintaining a working pressure of 5 mTorr and 100 W power. To complete the device, a top IZVO electrode (100 nm) was deposited onto the active switching layer using a metal shadow mask.

### Characterization

Transmittance spectra spanning the UV–visible wavelength range (200–800 nm) were acquired via UV–visible spectroscopy (Lambda-35, Perkin Elmer). The X-ray diffraction (XRD) patterns were obtained using an X-ray diffractometer (Rigaku, Smart Lab). For the IZO and V-doped IZO, X-ray photoelectron spectroscopy (XPS) measurements were taken with an X-ray photoelectron spectroscope (X-tool, ULVAC-PHI). The WF mapping images and spectra were generated using a Kelvin probe system (KP01, KP technology) and ultraviolet photoelectron spectroscopy (UPS). The sheet resistance was determined through a four-point probe system (CMT-SR1000N, Advanced Instrument Technology). The mobilities of all samples were assessed using a Hall effect measurement system (HMS-3500, Ecopia). Additionally, three-dimensional (3D) surface images were captured using atomic force microscopy (AFM) using the XE-100 model from Park Systems. The photovoltaic characteristics under forward bias were evaluated using a solar cell *I-V* test system (K3000, McScience) with a Keithley 2400 source measurement unit under AM 1.5 (100 mW cm^−2^) and an LED. The EQE spectra of the OSCs were acquired through the solar cell IPCE measurement system (K3100, McScience). The electrical and synaptic properties were measured using ArC ONE (UK) and Keithley 4200A instruments. Finally, the characteristics of the OLED were examined using the M6100 program (McScience, Republic of Korea) and SpectraScan PR-655 colorimeter.

## Results and Discussion

### Fabrication of mIZVO Electrode via Self-Cracking Template-Assisted Co-Sputtering Technique

**Fig. 1:**
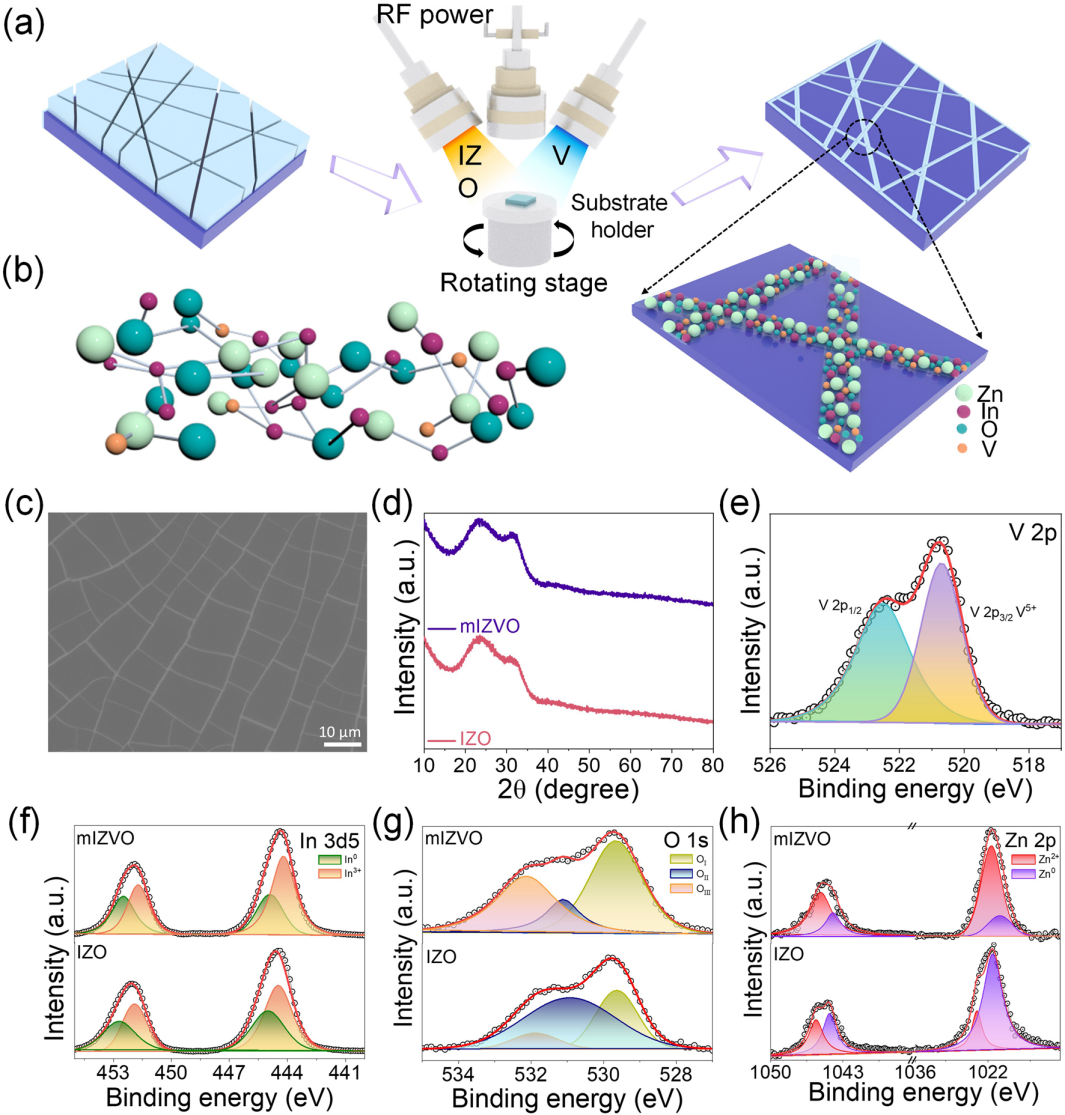
Fabrication and characterization of electrodes: **a** schematic of mesh electrode fabrication using the co-sputtering process. Mesh templates were first made by coating egg white on a flexible substrate with a glass rod and drying under ambient conditions. **b** Schematic illustration of the amorphous IZVO. **c** Top-view SEM image of mesh electrode fabricated through a self-cracking template. **d** XRD and **e** high-resolution XPS spectra of V 2*p*. Narrow scan XPS spectra: **f** In 3*d*5, **g** O 1*s*, and **h** Zn 2*p* before and after V doping

The mIZVO thin film was fabricated using a self-cracking template-assisted co-sputtering method for application as a flexible transparent electrode (Fig. 1a). First, a sacrificial layer of egg white was deposited on the flexible substrates using a glass rod. Subsequently, the assemblies were allowed to dry under ambient conditions, forming cracked templates. These cracked templates were then co-sputtered within a vacuum chamber, and the sacrificial egg white layer was dissolved in water to yield the final mIZVO. Further experimental details are outlined in the experimental methods section and Tables S1 and S2. Figure 1b presents a schematic diagram of the IZVO as a flexible transparent conducting electrode material. The morphology of the mIZVO electrode is revealed in the field emission scanning electron microscopy (FESEM) image presented in Fig. 1c, in which the random mesh network of the IZVO is displayed. A FESEM image of the self-cracking template after IZVO deposition is presented in Fig. S1a. Additionally, low- and high-magnification FESEM images of mIZVO are included in Fig. S1b, c. Figure S2a, b displays optical images that illustrate the cracked templates with and without oxygen plasma treatment. When subjected to oxygen plasma treatment, the PEN substrate became highly adhesive, allowing the materials to form a continuous and conductive network. Figure S2a reveals significant reductions in the size and number of cracks, indicative of crack coalescence and growth during the plasma treatment process. This phenomenon occurred because smaller cracks merged into larger ones due to stress relief and material reorganization, aligning with the material’s tendency to minimize overall energy by reducing the total crack surface area [41]. SEM images of mIZVO with and without oxygen plasma are presented in Fig. S3a, b, respectively. The observed enlargement in mesh size post-plasma treatment (Fig. S3) was attributed to several factors. Oxygen plasma activated the surface, enhancing adhesion and uniformity and facilitating the formation of a larger mesh structure. Additionally, this promoted material redistribution on the surface, creating more stable conductive pathways through increased surface energy and the removal of contaminants. Moreover, the treatment improved the uniformity of material deposition, resulting in a continuous network. Despite the larger mesh size, the conductivity was enhanced by establishing reliable and direct pathways for electron transport. Adhesion plays a pivotal role in guaranteeing the quality, performance, and durability of FTCEs. To assess the level of adhesion between the substrate and mesh electrode, we conducted a comprehensive suite of tests, including peel (scotch tape), scratch (eraser), shear (100 bending cycles), and ultrasonic exposure (sonication time of 5 min). Optical micrographs of the electrodes before and after the adhesion tests are presented in Fig. S4. Remarkably, no notable alterations in the mesh structure were observed following the adhesion tests, underscoring the effectiveness of oxygen plasma treatment for the development of continuous and conductive networks. As demonstrated in Fig. 1d, the XRD patterns of IZO and IZVO exhibit a distinctive broad amorphous hump, indicating the absence of crystalline features. The XRD analysis also confirmed the amorphous nature of the deposited IZO and IZVO thin films.

The chemical composition and bonding states of IZO and IZVO were analyzed using XPS, as depicted in Fig. S5a. Figure 1e illustrates the high-resolution spectra of V 2*p*, deconvoluted into V 2*p*_1/2_ and V 2*p*_3/2_. High-resolution XPS spectra of In 3*d*, O 1*s*, and Zn 2*p* for IZO and IZVO are illustrated in Fig. 1f–h. In Fig. 1f, the high-resolution spectra of the In 3*d* core levels exhibit a distinctive doublet structure, which was attributed to spin–orbit coupling. The two prominent peaks within this doublet corresponded to 3*d*_5/2_ and 3*d*_3/2_, respectively. Notably, dominant peaks assigned to In^3+^ were observed at 444.19 and 451.69 eV, while minor peaks attributed to In atoms in the reduced state (In^0^) were centered at 444.97 and 452.47 eV, which were separated by a consistent distance of 7.5 eV. These results are in good agreement with previously published reports [5, 42]. The O 1*s* core level spectrum displayed in Fig. 1g is divided into three distinct peaks: O_I_, O_II_, and O_III_. The O_I_ peak located at 529.78 eV corresponded to O^2−^ ions, while the O_II_ peak (positioned at 531.10 eV) corresponded to O^2−^ ions within oxygen-deficient regions (oxygen vacancies) (V^O^) [43] in the IZVO complex. The O_III_ peak at 532.01 eV was attributed to various bonds (such as O−H, O−C, and O−O), which originated from water and organic contaminants adsorbed onto the surface of the IZVO thin films [44]. As depicted in Fig. 1h, the high-resolution Zn 2*p* spectra for both IZO and m-IZVO display two sets of peaks for each spin–orbit doublet: 2*p*_3/2_ and 2*p*_1/2_. The lower-binding energy peaks in each doublet were assigned to zinc (Zn), while the higher-binding energy peaks were attributed to Zn^2+^. To assess alterations in the fitting peaks after doping, we calculated the area ratio of the deconvoluted peaks for elements in the IZO and IZVO (Fig. S5b). The O_II_/(O_I_ + O_II_) ratio serves as a measure of the relative density of V^O^ in the IZO and IZVO thin films. With an increase in V concentration, the O_II_/(O_I_ + O_II_) ratio significantly decreased from 44.28% to 40.95%, indicating a clear reduction in V^O^ density. Similar trends were observed for both In and Zn. By reducing the density of oxygen vacancies, we could reduce the carrier concentration, resulting in the customization of the electrical and optical properties of the IZO thin films. This interdependence was evident in the relationship between transmittance, resistivity, and the carrier concentration present in the thin films [22, 23].

### Optical Properties, Mechanical Flexibility, and WF Engineering of Electrodes

**Fig. 2:**
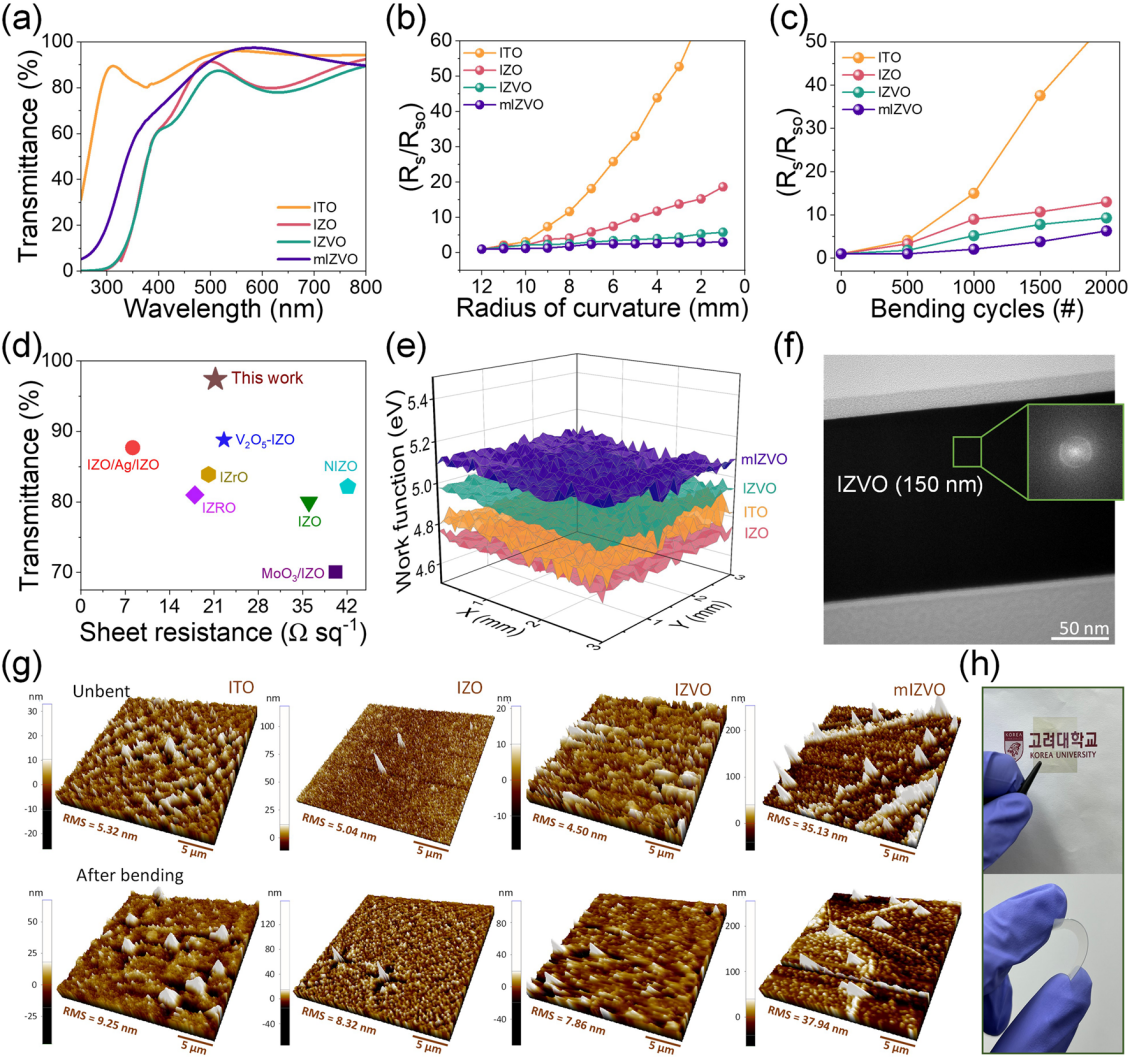
IZVO networks as a transparent flexible electrode: **a** UV–Vis transmittance spectra of different electrodes on PEN substrate. Variations in resistance of electrodes deposited on 200-µm-thick PEN substrate as a function of **b** radius of curvature and **c** bending cycles. **d** Comparison of experimentally measured transmittance and sheet resistance of the mIZVO electrode with recent publications. **e** WF of electrodes measured using the Kelvin probe measurement system. **f** Transmission electron microscopy image of the electrode. The thickness of the electrode was revealed as 150 nm (the inset displays the FFT of IZVO, indicating an amorphous nature). **g** Atomic force microscopy image of various electrodes before and after bending at a fixed radius of 2 mm. **h** Photographs of fabricated electrodes

Figure 2a confirms that the mIZVO electrode had an optical transmittance of 97.39% at a wavelength of 550 nm. It should be noted that the optical transmittance of the mIZVO electrode was marginally better than that of the ITO, IZO, and IZVO electrodes around 550 nm. The transmittance spectra recorded for various deposition parameters to optimize FTCE are summarized in Fig. S6. The transmittance value of over 97% for the electrode at 550 nm was determined by averaging multiple measurements. To ensure accuracy and consistency, we performed transmittance measurements 10 times at the same location on the electrode (Fig. S7a). Additionally, we addressed concerns about spatial variation by measuring transmittance at 10 different locations on the electrode, including densely packed and hollow areas (Fig. S7b). The spatial uniformity of the sheet resistance across the entire FTCE is crucial for assessing the performance accurately. To address this issue, we conducted an analysis by dividing the PEN substrate (2.5 cm × 2.5 cm) into 25 fragments and then measuring the sheet resistance across each fragment (Fig. S8). The average sheet resistance of mIZVO was revealed as 21.24 Ω sq^−1^. Additionally, the FoM was computed to analyze the trade-off between transmittance and R_s_ of the mIZVO electrode using Haacke’s equation [45]:

1 $$\text{FoM}=\frac{{T}^{10}}{{R}_{\text{s}}}$$

where *T* is the optical transmittance at 550 nm. The FoM of the fabricated mesh electrode was 3.61, which is significantly higher than previously reported values (Table S3). To enable a comprehensive assessment of the mechanical durability of mIZVO, we conducted bending tests at various radii of curvature and recorded the changes in *R*_S_ relative to the initial *R*_S_ (*R*_S0_) [*R*_S_/*R*_S0_], as depicted in Fig. 2b. The mIZVO FTCE exhibited a minimal change in R_S_/R_S0_, with only a 300% increase. In contrast, the IZO and IZVO FTCEs exhibited more significant changes in *R*_S_/*R*_S0_ (580% and 1865%, respectively) when the bending radius decreased to 1 mm. The ITO FTCE experienced notable fluctuations in *R*_S_/*R*_S0_ with changes in curvature. As displayed in Fig. 2c, the *R*_S_/*R*_S0_ of ITO increased considerably after 1000 bending cycles at a radius of 2 mm. Under the same conditions, IZO and IZVO demonstrated resistance changes of approximately 1300% and 930%, respectively. However, the mIZVO electrode displayed a comparatively smaller increase in resistance, exhibiting a 630% change after 2000 bending cycles at a bending radius of 2 mm. The small change in *R*_S_/*R*_S0_ for mIZVO, even after bending at a radius of 2 mm, was due to its unique mesh structure. This structure provides it with improved mechanical flexibility and more even stress distribution compared to the bulk electrodes. The large improvement in the bending performance of the mIZVO electrode was attributed to several factors. First, the unique structure of the mesh allows for enhanced flexibility and mechanical resilience. The interconnected nodes and struts distribute stress more evenly across the material, reducing the likelihood of fracture under bending conditions. Second, the use of IZVO material, which is known for its high elasticity and durability, further enhances the electrode’s ability to withstand repeated bending [5]. As a result, its electrical performance remains consistent, even when bent. Furthermore, the adhesion and uniformity are enhanced by the oxygen plasma treatment, which also contributes to the superior stability of the mIZVO electrode. This result highlighted its potential for application in flexible and transparent electronic devices. Figure 2d and Table S3 summarize the performance of the fabricated electrode with IZO-based electrodes and other transparent electrodes.

Figure 2e displays the Kelvin probe-mapped WF values of the ITO, IZO, IZVO, and mIZVO flexible FTCEs. Additionally, Fig. S9a provides an ultraviolet photoelectron spectroscopy analysis of the fabricated electrodes. Based on the secondary electron onset, the WF values of ITO, IZO, IZVO, and mIZVO were determined as 4.81, 4.71, 5.05, and 5.16 eV, respectively. The relatively higher WF value for the mIZVO FTCE would be expected to diminish the energy barrier between the electrode and the transport layer, facilitating efficient charge transfer. This increased WF value could be due to doping V in an atmosphere comprising argon and oxygen. Doping metal oxides with metals in an Ar/O_2_ atmosphere during sputtering can significantly enhance the WF [46], which can be attributed to the formation of surface dipoles and the reduction of oxygen vacancies. The Ar/O_2_ atmosphere enhances the incorporation of oxygen, which passivates defects and modifies the electronic structure. Feste et al. [47] demonstrated that these doping conditions increase the WF of metal oxides. The spectra obtained in the valence band region (Fig. S9b) indicate an intensity elongation up to the Fermi level. High-resolution transmission microscopy using fast Fourier transformation (FFT) was employed to confirm the amorphous nature of the IZVO. Figure 2f illustrates the diffused ring patterns in the images and the unorganized arrangement of atoms, highlighting the absence of nanoscale crystallinity. Additionally, the energy-dispersive X-ray spectroscopy (EDS) elemental mapping displayed in Fig. S10 confirmed the uniform deposition of the amorphous IZVO. This was evident from the even distribution of In, Zn, O, and V atoms, indicating the absence of phase separation or aggregation in the thin film structure. Figure 2g presents 3D surface plots of the electrodes. These were obtained via atomic force microscopy (AFM) in tapping mode over a 625 µm^2^ area for both pre- and post-bending conditions, with a bending radius of 2 mm over 1000 cycles. The root-mean-square (RMS) values before bending were 5.32, 5.04, 4.5, and 35.13 nm for ITO, IZO, IZVO, and mIZVO, respectively. Following 1000 bending cycles, the RMS value for ITO increased to 9.25, while IZO and IZVO exhibited a moderate change in sheet resistance. Remarkably, the mIZVO electrode exhibited the smallest variation of 8.01% in the RMS value after 1000 bending cycles at a bending radius of 2 mm. This minimal change in the sheet resistance of mIZVO compared to other FTCEs confirmed its superior mechanical stability, demonstrating its outstanding mechanical endurance as an FTCE. Figure S11 displays an AFM image of the mIZVO electrode and its corresponding height line profile, which remained at 150 nm. Additionally, Fig. 2h displays photographs of the fabricated mIZVO electrode.

### Device-Level Validation of Fabricated Electrode

#### OSCs

**Fig. 3:**
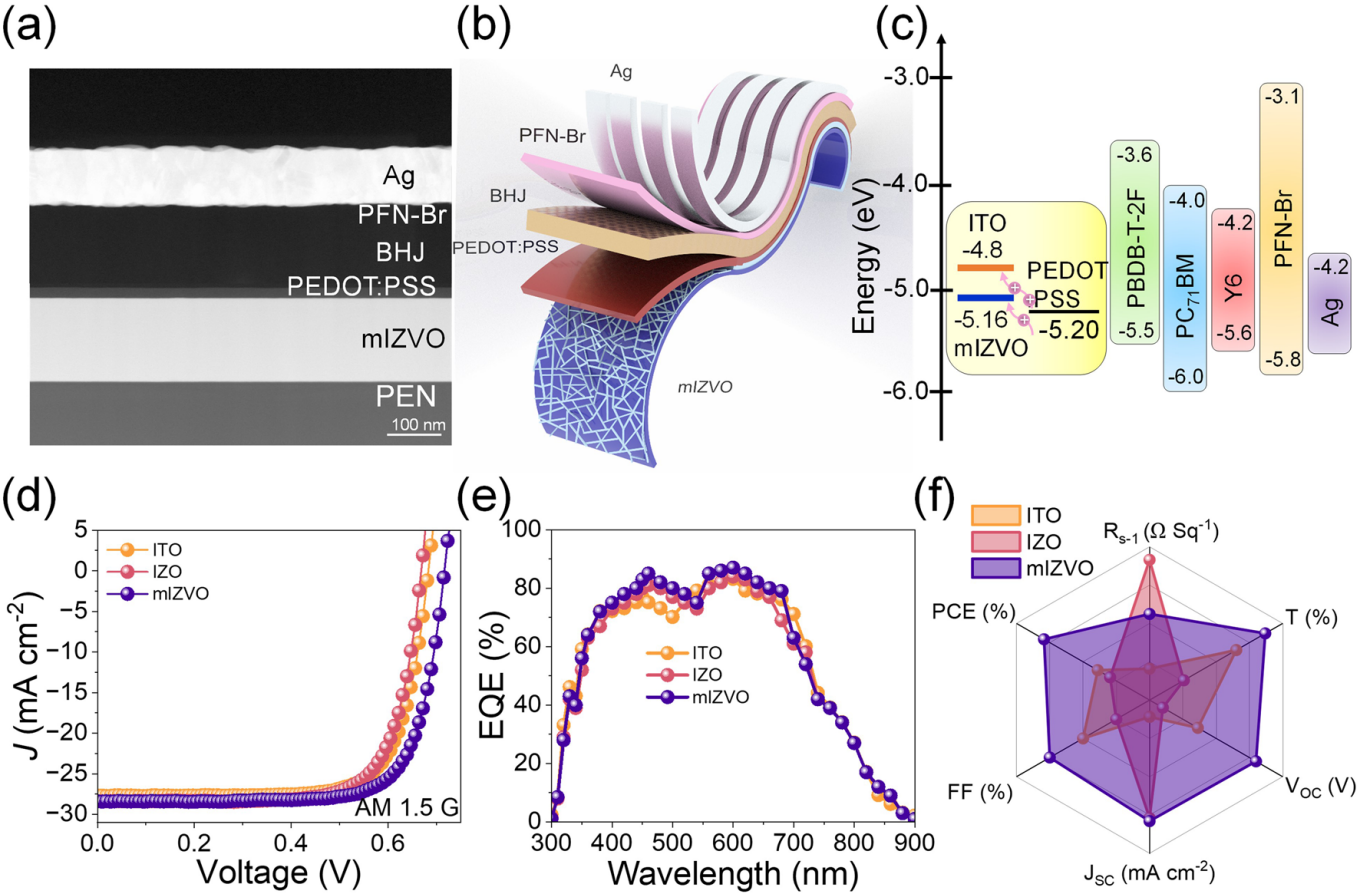
mIZVO as a transparent electrode for flexible organic solar cells: **a** cross-sectional TEM image of the fabricated organic solar cell. **b** Schematic of the organic solar cell with mIZVO as a flexible electrode. **c** Energy band alignment of the organic solar cell. *J-V* characteristics of the cell under **d** one sun (AM 1.5 G) and **e** EQE spectra of the best-performing cells utilizing various electrodes. **f** Radar plot comparing features of the electrodes and their photovoltaic performance

OSCs have gained significant attention due to their light weight, flexibility, semi-transparency, and large-scale processability. Moreover, with power conversion efficiencies exceeding 19%, these technologies demonstrate great potential for near-future commercial applications [48]. FTCEs play a crucial role in the architecture of flexible solar cells because they directly influence cell performance. To establish an effective ohmic contact, the electrode material must have high electrical conductivity, excellent optical transmittance, compatible band alignment, and a WF selected to operate seamlessly with the adjacent layers [49]. Accordingly, mesh electrodes and metal nanowires (MNWs) are attracting extensive attention as FTCEs in OSCs. However, the rapid degradation of photovoltaic performance due to the junction resistance and optical haze of MNW-based electrodes limit their use as FTCEs [50]. Flexible OSCs featuring a conventional PEN/mIZVO/PEDOT:PSS/photoactive layer/PFN-Br/ Ag structure were successfully fabricated using PM6:Y6:PC_71_BM (1:1:0.2) as the photoactive layer material. Detailed information on the fabrication process is provided in Fig. S12 and the experimental section. To confirm the continuity and integration of the OSC layers, a cross-sectional HRTEM analysis of an OSC with an mIZVO electrode was conducted, which demonstrated that there was an excellent interface of different layers without any intermixing, as delineated in Fig. 3a. A schematic illustration of the mIZVO-based OSC is presented in Fig. 3b, while Fig. 3c depicts the energy band diagram for the corresponding device. The current–voltage (*J-V*) curves of the OSCs were measured under 1 sun illumination conditions (100 mW cm^−2^, AM 1.5G), as displayed in Fig. 3d. A control device employing an ITO electrode demonstrated a *J*_SC_ of 27.71 mA cm^−2^, a *V*_OC_ of 0.689 V, an FF of 69%, and a PCE of 13.17%. In comparison, the IZO-based OSCs exhibited moderate performance with a PCE of 12.89%, which was primarily attributed to the low FoM of pristine IZO electrodes. The best-performing cell based on the mIZVO electrode achieved a PCE of 14.38%. The introduction of V doping in the IZO and mesh engineering resulted in an enhancement in terms of PCE, which was attributed to the ultra-transparency of the electrodes, the high conductivity, and favorable WF alignment with the adjacent PEDOT:PSS hole transport layer. The external quantum efficiency (EQE) spectra of the devices were then examined to evaluate their photoresponse over the entire absorption region (Fig. 3e). The PCE values of the solar cells were calculated using the following formula:

2 $$\text{PCE}=\frac{{V}_{\text{oc}}\times {I}_{\text{sc}}\times \text{FF}}{{P}_{\text{in}}}\times 100$$

We observed that devices based on highly transparent FTCEs exhibited a remarkable photoresponse in the 400–800 nm region, which was expected. A radar plot summarizing the electrode properties and device performance is presented in Fig. 3f. In addition, the performance of the fabricated electrode and its photovoltaic output characteristics are compared with existing electrodes in Table S4. These results indicated that the mIZVO electrode has great potential for fabricating highly efficient organic solar cells.

#### OLEDs

**Fig. 4:**
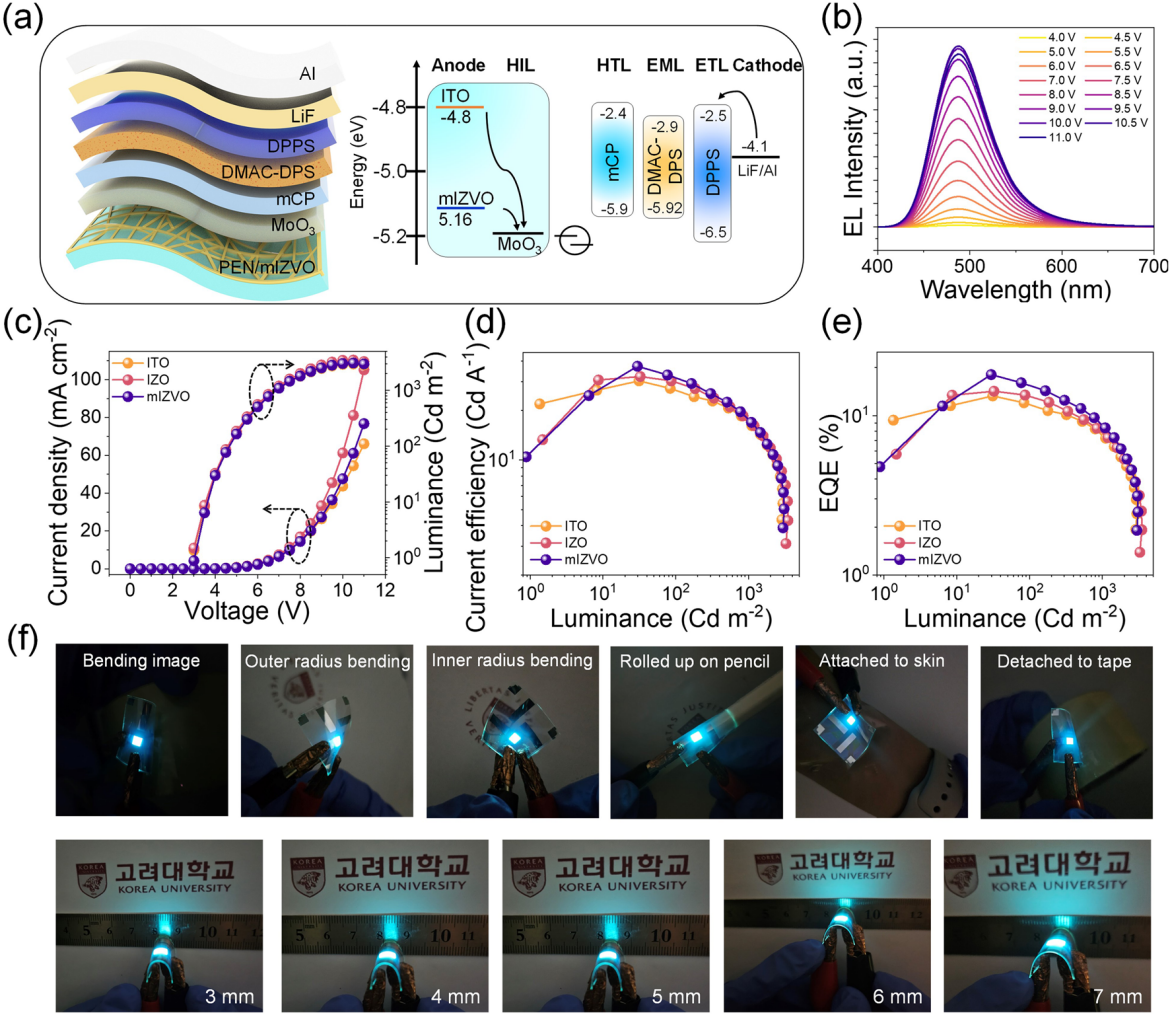
mIZVO as a transparent electrode for OLEDs: **a** schematic of OLED device with energy band alignment. **b** EL intensity under different voltages for mIZVO-based OLED devices. **c** Current density–voltage–luminance, **d** current efficiency vs. luminance, and **e** luminance-EQE characteristics of ITO, IZO, and mIZVO electrode-based OLEDs. **f** Optical images of flexible OLEDs with mIZVO anode at different bending curvatures

Over the past decade, OLED displays have emerged as the predominant technology for television and smartphone screens, driven by their superior performance metrics and aesthetic benefits [51, 52]. To explore the performance of the mIZVO FTCEs, OLEDs were fabricated with 150-nm-thick mIZVO, and their performance was compared with ITO and IZO electrode-based OLEDs. The fabrication process involved stacking a series of organic materials, followed by depositing an Al top electrode. For more information on the fabrication process, please refer to the experimental section. Figure 4a depicts schematics of the OLED using FTCEs and an energy-level diagram of the device. The higher WF of the electrode contributed to reducing the potential barrier difference with the hole injection layer (HIL), enhancing the hole injection rates and facilitating exciton recombination. The mIZVO devices exhibited stable electroluminescence (EL) spectra at a constant wavelength of 485 nm with no noticeable shift, even under continuous operation, as depicted in Fig. 4b. The current density–voltage–luminescence (*J-V-L*) plots are displayed in Fig. 4c. Although all the devices had a turn-on voltage of 3 V, the mIZVO-based device exhibited a slight decrease in maximum luminescence value, which was potentially due to inadequate electrode coverage. Interestingly, the current injection in mIZVO was superior to both IZO and ITO (Fig. 4d), which was attributed to the reduced injection barrier, as depicted in Fig. 4a. Figure 4e displays a plot of EQE against the luminescence characteristic spectra. The EQE of the mIZVO-based OLED device was 18.06%, which was higher than both IZO (14.22%) and the reference ITO (13.29%). This result was due to the good alignment of energy levels and the high transparency of mIZVO. Figure 4f presents photographs of the mIZVO-based OLED under different bending conditions. The video file included in the supplementary material confirms the device’s stability during bending. The performance of the fabricated electrodes in terms of turn-on voltage, external quantum efficiency, and luminescence was then compared with existing electrodes, as listed in Table S5. The device using mesh electrodes exhibited a higher EQE compared to the other tested devices. All the findings suggested that OLEDs employing IZVO mesh electrodes hold significant promise for the production of flexible, large-area, high-performance electronics, and sensors.

#### Flexible Transparent Memristors for Neuromorphic Computing

**Fig. 5:**
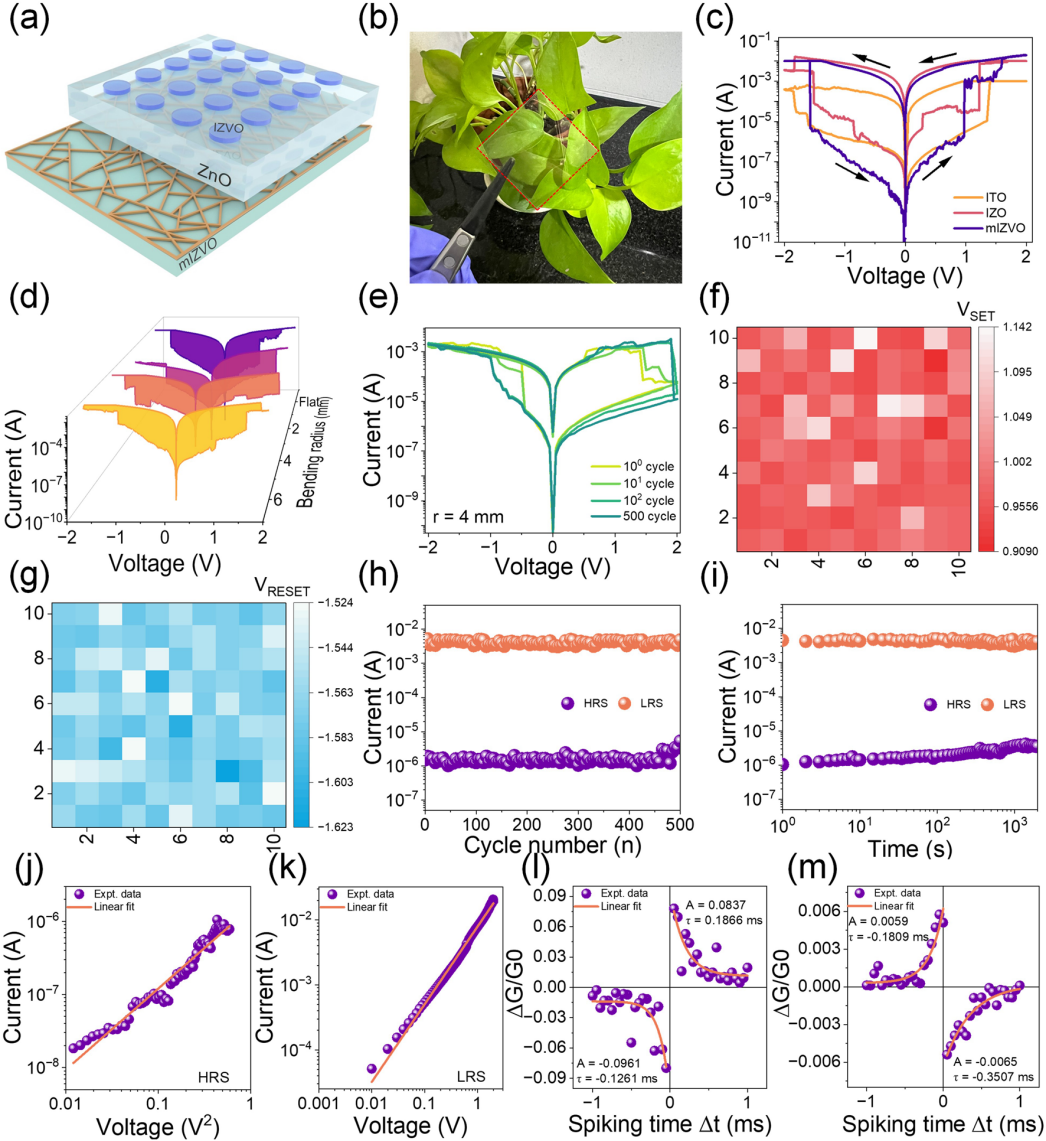
mIZVO as a transparent electrode for FT memristors: **a** typical device structure and **b** photograph of the FT memristor. **c** Typical *I-V* characteristics of the memristor with ITO, IZO, and mIZVO as flexible bottom electrodes. **d** *I-V* characteristics of a memristor when subjected to mechanical deformation with varying bending radii. SET and RESET voltage distribution of **e** *I-V* characteristics of the FT memristor device tested for different cycles at fixed *r* = 4 mm. Distribution of **f** SET and **g** RESET voltages for 100 samples. **h** Endurance of HRS and LRS over 500 switching cycles. **i** Retention of HRS and LRS, demonstrating an ON/OFF ratio greater than 10^3^. Fitted *I-V* curve of positive bias for **j** HRS and **k** LRS in double logarithmic scale. Implementation of STDP with millisecond-scale time windows in synapse mimicking the **l** antisymmetric Hebbian learning rule and **m** antisymmetric anti-Hebbian learning rule

With the rapid advancement of intelligent, transparent, flexible, and wearable electronic devices, the limited transmittance of conventional memristor devices does not meet the demands of various futuristic applications, such as AI-enabled smart home systems, intelligent health monitoring, and IoT-based wearable edge devices. To broaden the range of applicability of the fabricated FTCEs, we employed mIZVO to develop FT-type memristor devices. Figure 5a presents a schematic diagram of an FT memristor, with mIZVO as the bottom electrode, ZnO as the switching layer, and IZVO as the top electrode. To understand the transparency of the device, a photograph of the developed memristor is displayed in Fig. 5b. The optical transmittance (Fig. S13) over the visible range was over 90% at a wavelength of 550 nm. Implementing mIZVO as the bottom electrode in a memristor can improve transparency. Figure 5c displays the *I-V* characteristics of FT memristor devices with ITO/ZnO/ITO, IZO/ZnO/IZO, and mIZVO/ZnO/IZVO structures. All the devices demonstrated bipolar resistive switching behavior, although the mIZVO-based memristor exhibited superior performance compared to the ITO and IZO-based devices. The mesh electrode structure of the mIZVO device potentially enhanced memory performance by offering a larger surface area and reduced resistive losses, improving charge injection and extraction. This result indicates that it would be particularly advantageous for high-performance applications and integration onto flexible substrates. Consequently, we selected the mIZVO-based memristor for further testing. When subjected to a bipolar bias, the mIZVO-based device transitioned from a high resistance state (HRS) to a low resistance state (LRS) at a set voltage (*V*_SET_) of 0.97 V. Similarly, when a reset voltage (*V*_RESET_) of −1.57 V was reached, the memristor seamlessly transitioned back to the HRS. The mechanical flexibility of the fabricated device was tested by measuring the switching performance under different bending conditions, as depicted in Fig. 5d. Compared to its initial state (prior to mechanical deformation), the device exhibited no noticeable degradation in terms of ON/OFF ratio, even when subjected to a bending radius of 1.0 mm. The switching performance of the device over multiple switching cycles at a fixed bending radius of 4 mm is presented in Fig. 5e. The distributions of *V*_SET_ and *V*_RESET_ to indicate device-to-device variability are displayed in Fig. 5f, g, respectively. Here, very low switching variations were observed during the device-to-device operations, demonstrating the excellent reliability of the mIZVO/ZnO/IZVO memristor. Figure 5h highlights the cyclic stability of the FT memristor. Although there were slight fluctuations in HRS and LRS, the switching behavior of the FT memristor was maintained over 500 switching cycles, indicating that it would be suitable for flexible and transparent non-volatile memory applications [53]. The retention performance of the FT memristor is presented in Fig. 5i, where it is evident that the device maintained a high ON/OFF ratio exceeding 10^3^ s with no apparent degradation in LRS and HRS. The following pulse conditions were used for the endurance test: (i) write voltage: ± 1.5 V, (ii) read voltage: 0.5 V, (ii) pulse width: 20 μs, and (iv) pulse period: 40 μs. In contrast, a constant voltage stress was applied over time for the retention test. To gain a deeper insight into the resistance-switching mechanism of the FT memristor, the positive voltage portions of the *I–V* curves for both HRS and LRS were represented in double logarithmic coordinates, as depicted in Fig. 5j, k, respectively. The Child’s square law and Ohmic current conduction models were dominant in the HRS and LRS regions, respectively. The device demonstrated forming-free operation due to the abundant intrinsic charge carriers within the switching layer. Furthermore, the ionized state of In (represented by In^3+^ cations) migrated to the active switching layer, where it combined with electrons to create In atoms, ultimately establishing a permeation network that functioned as an efficient carrier pathway. Consequently, the device operated effectively without requiring any forming operation. Figure S14 presents the detailed switching mechanism of the FT memristor based on the formation and rupture of the conduction filament(s).

When employed as synaptic devices, memristors can mimic biological synapses, enabling the construction of artificial neural networks that can significantly enhance neuromorphic computing capabilities [54, 55]. In conventional nervous systems, the typical neuron structure originates from interconnections between the pre- and post-neurons, as depicted in Fig. S15a. An electrical impulse prompts the presynaptic neuron to release neurotransmitters into the synaptic cleft. These neurotransmitters subsequently diffuse through the synaptic cleft and attach to receptors located on the post-synaptic neuron, facilitating the transmission of neural signals [56]. The strength of these connections between the neurons is referred to as the synaptic weight [57, 58]. Figure S15b displays the changes in resistance when the device was programmed by a series of 20 positive and 20 negative pulses. Here, we observed a gradual strengthening or weakening of the synaptic weight with an increasing number of positive or negative pulses, respectively. This corresponded with the potentiation (learning) or depression (forgetting) processes within the synapse. These results confirmed that the presented FT memristor could mimic the basic learning and forgetting functions of biological synapses. In the next stage, we mimicked the complex learning rules of biological synapses with the help of an FT memristor. In particular, we mimicked the spike-time-dependent plasticity (STDP)-based Hebbian learning rules. STDP is a mechanism through which the strength of connections between two or more neurons can be adjusted [59]. Long-term potentiation (LTP) refers to enhancing the synaptic weight change when the presynaptic neuron stimulus appears before the post-synaptic neuron stimulus. In contrast, when the post-synaptic stimulus appears first, immediately followed by the presynaptic stimulus, this suggests long-term depression (LTD). In neuroscience, Hebbian learning-based STDP rules hold significant importance and can be replicated by modifying the time intervals between pre- and post-synaptic spikes. In this study, we successfully mimicked the following four important rules with the help of the FT memristor: antisymmetric Hebbian learning, antisymmetric anti-Hebbian learning, symmetric Hebbian, and symmetric anti-Hebbian. These rules are delineated in Figs. 5l, m and S15c, d, respectively. The typical spike scheme to mimic STDP rules is displayed in Fig. S16. The data acquired from the antisymmetric Hebbian and antisymmetric anti-Hebbian learning rules were fitted with an exponential function (Eq. 3). Additionally, the learning functions for both the symmetric Hebbian and symmetric anti-Hebbian learning rules were modeled using a Gaussian function (Eq. 4) [19].

3 $$\Delta \text{W}=\text{A}\times {\text{e}}^{(\frac{-\Delta \text{t}}{\uptau })}+ \Delta {\text{w}}_{0}$$

4 $$\Delta \text{W}=\text{A}\times {\text{e}}^{\left(\frac{-\Delta {\text{t}}^{2}}{{\uptau }^{2}}\right)}+ \Delta {\text{w}}_{0}$$

Here, A represents the scaling factor, *τ* is the time constant, and ΔW denotes the alteration in the synaptic weight. The obtained values of the scaling factor and time constant are presented in the inset of Figs. 5l, m and S15c, d*.* These experimental findings indicated that the FT memristor could operate similarly to an artificial synaptic device, rendering it a promising candidate for applications in neuromorphic computing. A comparison of the non-volatile memory and synaptic performance between the fabricated and existing memory devices is summarized in Table S6. These results confirmed that the presented FT memristor has excellent resistive switching properties and represents a potential device for non-volatile memory and synaptic learning applications.

## Conclusions

In this study, we developed a highly efficient mesh IZVO electrode by using a self-cracking template. This electrode was designed to be ultra-transparent, with a transparency level of 97.39%. It also exhibited a low sheet resistance of 21.24 Ω sq^−1^ and an elevated WF of 5.16 eV. Implementation of this electrode in OSCs resulted in a PCE of 14.38%. Furthermore, the mIZVO-based OLEDs achieved an EQE of 18.06%. Interestingly, the FT memristors based on mesh IZVO electrodes exhibited low device-to-device and cycle-to-cycle variability, good non-volatile memory performance, and successfully mimicked basic and advanced synaptic learning properties. This outstanding performance was attributed to the ultra-transparency, low surface roughness, and aligned WF of the fabricated electrodes. A performance comparison with the existing literature suggested that our flexible mIZVO electrode has superior performance and can be integrated into various practical real-world applications. Furthermore, the enhanced properties of the mIZVO electrodes (such as increased flexibility and transparency) are critical for next-generation flexible optoelectronics. Overall, our findings suggest that mIZVO is a promising electrode for futuristic multifunctional flexible optoelectronic devices.

## Supplementary Information

Below is the link to the electronic supplementary material.

Supplementary file 1

Supplementary file 2
