# Supplementary material for: Ultra-Transparent and Multifunctional IZVO Mesh Electrodes for Next-Generation Flexible Optoelectronics
Source: Nanomicro Lett. 2024 Sep 26;17:12. doi: 10.1007/s40820-024-01525-y (PMC11427630; doi:10.1007/s40820-024-01525-y)
Supplement: Supplementary file 1 [file 40820_2024_1525_MOESM1_ESM.docx]

Supporting Information for

**Ultra-Transparent and Multifunctional IZVO Mesh Electrodes for Next-Generation Flexible Optoelectronics**

Kiran A. Nirmal^1^, Tukaram D. Dongale^2^, Atul C. Khot^1^, Chenjie Yao^1^, Nahyun Kim^1^ and Tae Geun Kim^1,^*

^1^School of Electrical Engineering, Korea University, Anam-ro 145, Seongbuk-gu, Seoul

^2^Computational Electronics and Nanoscience Research Laboratory, School of Nanoscience and Biotechnology, Shivaji University, Kolhapur 416004, India

*Corresponding author. E-mail: [tgkim1@korea.ac.kr](mailto:tgkim1@korea.ac.kr) (Tae Geun Kim)

**Supplementary Figures and Tables**

**Fig. S1 a** FESEM image of the self-cracking template after deposition of IZVO. **b** Low and **c** high magnification SEM images of mIZVO

**Fig. S2** Optical microscopy images showcasing the cracked template **a** with and **b** without oxygen plasma treatment. The adhesion appears to be enhanced following the treatment judging from the increase in pattern size, which is probably due to either dry etching or the surface modification effect. The optical images were captured at the same magnification using a 50× objective lens

**Fig. S3** SEM images of mIZVO **a** before and **b** after oxygen plasma treatment, prior to being coated with egg white

**Fig. S4** Optical microscopic images of mesh electrodes before and after **a** peel test (using 3M scotch tape), **b** scratch test (eraser), **c** shear test (100 bending cycles), and **d** ultrasonic exposure test (sonication time 5 min). All the optical images were captured using a 50× objective lens

**Fig. S5 a** XPS survey spectra of the IZO and IZVO thin films. **b** Area ratios calculated using the area of deconvoluted spectra of In, O, and Zn

**Fig. S6** Transmittance spectra of the IZVO thin films with variation in deposition parameters: **a** vanadium doping concentration, **b** oxygen partial pressure, **c** process pressure, and **d** electrode thickness

**Fig. S7** Transmittance spectra of mIZVO electrode measured **a** for 10 cycles and **b** at 10 different positions

**Fig. S8** Area-to-area variability of sheet resistance of mIZVO electrode on PEN substrate

**Fig. S9 a** UPS spectra of electrodes around the secondary electron cut-off (measured at 21.22 eV). **b** Valence band maxima of electrodes by UPS

**Fig. S10** TEM image of mIZVO electrode and EDS mapping images of each element

**Fig. S11** AFM image of mesh electrode with height line profile

**Fig. S12** Schematic illustration of organic solar cell fabrication protocol

**Fig. S13** Optical transmittance of FT memristor device. The inset shows a photograph of an FT memristor device

**Fig. S14** The possible switching mechanism of FT memristive device at **a** no bias, **b** LRS, and **c** HRS

Based on the results of conduction model fitting, the mechanisms of filament growth and destruction are elucidated in Fig. S14. When a positive bias was applied to the top electrode, the accumulation of oxygen vacancies within ZnO facilitated the formation of a conductive path linking the top and bottom electrodes [S1, S2]. Furthermore, the ionized state of In (represented by In^3+^ cations) migrated to the active switching layer, where it combined with electrons to create In atoms. Continuous ionization and migration of In^3+^ cations aided in filament formation, with the smaller ionic radii of In^3+^ facilitating path assessment through the switching layer [S3]. Conversely, during the reset process, applying a negative bias to the top electrode initiated the movement of In^3+^ ions back to the top electrode. Additionally, the filament formed by the aggregation of oxygen vacancies ceased due to joule heating. In this manner, the formation and rupture of the conductive filament governed resistive switching.

**Fig. S15 a** Schematic of a biological synapse. **b** Potentiation and depression, **c** symmetric Hebbian (SH), and **d** symmetric anti-Hebbian (SAH) learning rules mimicked by a transparent flexible device

**Fig. S16** Spike applied to measure **a** symmetric Hebbian (SH), **b** symmetric anti-Hebbian (SAH), **c** asymmetric Hebbian (ASH), and **d** asymmetric anti-Hebbian (ASAH) learning rules by a transparent flexible device

**Table S1** Magnetron sputtering deposition conditions

| Electrode | | Target | Working pressure  (mTorr) | Forward power  (W) | Base pressure  (Torr) | Gas | Gas flow | Deposition rate (nm s^-1^) |
| --- | --- | --- | --- | --- | --- | --- | --- | --- |
| ITO | ITO | | 2 | 70 | 2 × 10^−6^ | Ar | 40 | 0.1408 |
| IZO | IZO | | 2 | 100 | 2 × 10^−6^ | Ar/O_2_ | 20/0.3 | 0.1923 |
| IZVO | IZO/V | | 2 | 100/8 | 2 × 10^−6^ | Ar/O_2_ | 20/0.3 | 0.2083 |

**Table S2** Performance of IZVO thin films with various process parameters

| Parameters |  | Rs (Ω/sq) | µ _Hall_ (cm^2^/Vs) | Conductivity (/Ω cm) | Transmittance @ 550 nm | Work function  (eV) |
| --- | --- | --- | --- | --- | --- | --- |
| Process pressure  (mTorr) | 2 | 22.85 | 6.45 | 87.98 | 94.78 | 5.05 |
|  | 5 | 30.25 | 8.30 | 68.40 | 89.80 | 5.05 |
|  | 10 | 41.78 | 2.84 | 48.05 | 85.19 | 5.03 |
|  | 30 | 56.23 | 4.91 | 37.05 | 88.41 | 4.96 |
| Oxygen (O_2_) partial  pressure  (sccm) | 0.2 | 19.86 | 7.92 | 100.03 | 85.08 | 4.95 |
|  | 0.3 | 22.85 | 6.45 | 87.98 | 95.64 | 5.05 |
|  | 0.4 | 21.09 | 5.15 | 93.29 | 83.07 | 5.15 |
|  | 0.5 | 23.15 | 8.8 | 85.94 | 91.42 | 5.17 |
| Film thickness (nm) | 50 | 91.5 | 7.5 | 101 | 84.51 | 4.73 |
|  | 75 | 49.05 | 6.3 | 97.25 | 83.20 | 4.84 |
|  | 100 | 35.98 | 5.2 | 93.55 | 89.23 | 4.89 |
|  | 125 | 28.05 | 8.1 | 87.95 | 96.11 | 4.97 |
|  | 150 | 23.57 | 6.45 | 80.98 | 94.76 | 5.05 |
|  | 200 | 16.67 | 6.51 | 68.98 | 81.81 | 5.14 |
| Vanadium doping power | 0 | 23.95 | 6.59 | 84.72 | 94.92 | 4.81 |
|  | 4 | 22.40 | 6.78 | 88.78 | 93.04 | 4.93 |
|  | 8 | 22.86 | 6.45 | 87.98 | 95.18 | 5.05 |
|  | 12 | 30.12 | 4.14 | 67.89 | 90.24 | 5.07 |
|  | 16 | 39.33 | 2.55 | 53.03 | 76.33 | 5.15 |

**Table S3** Comparison of optimized mIZVO TCEs and existing flexible transparent electrodes in terms of transmittance, sheet resistance, FoM values, and mechanical flexibility

| Electrodes | Transmittance  [%, 550 nm] | Sheet resistance  [Ω sq^-1^] | FoM [Ω^-1^] | Mechanical stability^a)^ | Refs. |
| --- | --- | --- | --- | --- | --- |
| MXene | 82 | 108 | 0.12 | - | [S4] |
| ITO mesh | 70.5 | 151 | 0.02 | 6.85 mm, 1000 cycles | [S5] |
| Au/Graphene | 74.6 | 24.6 | 0.22 | 5 mm, 1800 cycles | [S6] |
| Ag mesh | 84.4 | 12.50 | 1.46 | 10 mm, 10000 cycles | [S7] |
| Graphene/CNTs /PEDOT:PSS | ≥80 | 43.2 | 0.25 | 10 mm, 1000 cycles | [S8] |
| Ag NW | ≥80 | 15.2 | 0.71 | 5 mm, 10000 cycles | [S9] |
| AgNW:ZnO | 84.5 | 20.5 | 0.91 | 3 mm, 1500 cycles | [S10] |
| mNIZO | 95.1 | 28.4 | 2.12 | 2 mm, 2000 cycles | [S11] |
| V_2_O_5_ Graded IZO | 82.15 | 42.14 | 0.29 | 10 mm, 10000 cycles | [S12] |
| Al mesh | 80 | 24 | 0.45 | 1 mm, 500 cycles | [S13] |
| Ag mesh | 93.7 | 1.4 | 3.7 | 4mm, 800 cycles | [S14] |
| Cu mesh | 93 | 13.4 | 3.6 | 9 mm, 600 cycles | [S15] |
| Ag mesh | 86.39 | 6.08 | 3.8 | 1 cm, 1000 cycles | [S16] |
| Cu-Ag mesh | 87.2 | 9.3 | 2.7 | 5 mm, 1000 cycles | [S17] |
| mIZVO | 97.39 | 21.24 | 3.61 | 2 mm, 2000 cycles | This work |

**Table S4** Comparison between fabricated and existing FTCEs for OSCs

| FTCE | Rsh  (Ω/□) | T (%)  @550nm | Jsc  (mA cm^−2^) | Voc  (V) | FF  (%) | PCE  (%) | Refs. |
| --- | --- | --- | --- | --- | --- | --- | --- |
| Graphitic carbon film | 1.7 × 10^3^ | - | 4.76 | 0.55 | 45.16 | 1.19 | [S18] |
| Zn:MXene/Ag/MXene | 9.7 | 84 | 33.49 | 0.641 | 71.38 | 13.86 | [S19] |
| AgNW/MXene/Gr | 18.1 | 88.1 | 7.44 | 0.561 | 25.83 | 1.12 | [S20] |
| GO/AgNW | 18 | 87 | 19.84 | 0.68 | 57 | 7.62 | [S21] |
| Gr-AgNWs | 103 | 66 | 21.07 | 0.77 | 58 | 9.45 | [S22] |
| NIZO | 250 | 91.5 | 0.9 | 0.305 | 22.1 | 0.21 | [S23] |
| V_2_O_5_-graded IZO | 42.15 | 82.15 | 8.104 | 0.571 | 59.48 | 2.753 | [S12] |
| ITO | 14.1 | 93.04 | 27.71 | 0.689 | 69 | 13.17 | This work |
| IZO | 28.30 | 85.16 | 28.37 | 0.668 | 68 | 12.89 | This work |
| mIZVO | 21.24 | 97.39 | 28.39 | 0.724 | 70 | 14.38 | This work |

**Table S5** Performance comparison between fabricated and existing FTCEs for OLEDs

| Substrate | Electrode | V_on_ [V]*^a)^* | CE_max_  [cd/A] *^b)^* | PE_max_  [Im/W] *^c)^* | η_ext, max_  [%]*^d)^* | L_max_  [Cd/m^2^]*^e)^* | λ_EL_ [nm]*^f)^* | Refs. |
| --- | --- | --- | --- | --- | --- | --- | --- | --- |
| PET | Ag NW microgrid | 2.9 | 27.7 | - | - | - | 510 | [S24] |
| PET | PEDOT:PSS/RM | 4.1 | 28.1 | 25.6 | - | - | - | [S25] |
| PET-PVA | Cu NW | 3.5 | 28.3 | - | - | 14130 | - | [S26] |
| PEN | Te–W | 3.2 | 29.1 | 24.7 | 15.7 | 2150 | 484 | [S27] |
| CPI | IGTO/APC/IGTO | 3.0 | - | - | 7.8 | 2000 | 460 | [S28] |
| PEN | Au mesh | 3.4 | - | 1.4 | 3.6 | 4000 | - | [S29] |
| NOA | IZO mesh | 4 | - | - | 11.7 | 1300 | 480 | [S30] |
| NOA | Ti/IZO mesh | 4 | - | - | 13.2 | 1400 | 480 | [S30] |
| PEN | mIZVO | 3.0 | 37.14 | 29.17 | 18.06 | 3086.7 | 480 | This work |

a) Turn on voltage; b) Maximum CE; c) Maximum PE; d) Maximum EQE; e) Maximum luminance; f) EL peak wavelength

**Table S6** Performance comparison between mIZVO FTCE-based and existing memristors based on oxide electrodes

| Device structure | V_SET_/  V_RESET_ | Endurance (cycles) | Retention  (s) | Flexibility | Synaptic  learning | Refs. |
| --- | --- | --- | --- | --- | --- | --- |
| ITO/ZnO/ITO | +2, −2 | - | - | - | PD, PPF | [S1] |
| ITO/ZnO/ITO | +2, −1.5 | 100 | - | - | - | [S31] |
| ITO/TaOx/ITO | +4, −4 | 800 | 10^3^ | 10^3^ cycles at 4 mm | - | [S3] |
| AZO/NiO/AZO/muscovite | +3, −4 | 1000 | 10^4^ | 10^3^ cycles at 6.5 mm | - | [S32] |
| ITO/boehmite/ITO | +2, −3 | 300 | 10^4^ | 250 cycles at 3.82 mm | - | [S33] |
| AZO/ZnO/ITO | +5, −2.5 | 100 | - | - | - | [S34] |
| GZO/ZnO_2_/ZnO/ITO | +5, −2.5 | 100 | 7×10^3^ | - | PD | [S35] |
| IZVO/ZnO/mIZVO | +0.97, −1.57 | 500 | ~10^4^ | 10^3^ cycles at 4 mm | PD, STDP | This work |

**Supplementary References**

[S1] P.S. Subin, K.J. Saji, M.K. Jayaraj, Plasticity and learning behavior emulated in a ZnO based transparent artificial synapse, Thin Solid Films **737**, 138924 (2021). <https://doi.org/10.1016/j.tsf.2021.138924>

[S2] M. Noh, D. Ju, S. Cho, S. Kim, The enhanced performance of neuromorphic computing hardware in an ITO/ZnO/HfOx/W bilayer-structured memory device, Nanomater. **13**, 2856 (2023). <https://doi.org/10.3390/nano13212856>

[S3] S. Rajasekaran, F.M. Simanjuntak, D. Panda, S. Chandrasekaran, R. Aluguri et al., Fast, Highly Flexible, and transparent TaOx-based environmentally robust memristors for wearable and aerospace applications, ACS Appl. Electron. Mater. **2**, 3131–3140 (2020). <https://doi.org/10.1021/acsaelm.0c00441>

[S4] S. Ahn, T.-H. Han, K. Maleski, J. Song, Y.-H. Kim et al., A 2D titanium carbide mxene flexible electrode for high-efficiency light-emitting diodes, Adv. Mater. **32,** 2000919 (2020). <https://doi.org/10.1002/adma.202000919>

[S5] K. Sakamoto, H. Kuwae, N. Kobayashi, A. Nobori, S. Shoji et al., Highly flexible transparent electrodes based on mesh-patterned rigid indium tin oxide, Sci. Rep. **8**, 2825 (2018). <https://doi.org/10.1038/s41598-018-20978-x>

[S6] Y. Chen, X.-Y. Fu, Y.-Y. Yue, N. Zhang, J. Feng et al., Flexible and transparent supercapacitor based on ultrathin Au/graphene composite electrodes, Appl. Surf. Sci. **467**, 104–111 (2019). <https://doi.org/10.1016/j.apsusc.2018.10.093>

[S7] K.-W. Seo, Y.-J. Noh, S.-I. Na, H.-K. Kim, Random mesh-like Ag networks prepared via self-assembled Ag nanoparticles for ITO-free flexible organic solar cells, Sol. Energy Mater. Sol. Cells **155**, 51–59 (2016). <https://doi.org/10.1016/j.solmat.2016.04.056>

[S8] T. Wang, Y.-Z. Wang, L.-C. Jing, Q. Zhu, A.S. Ethiraj et al., Novel biodegradable and ultra-flexible transparent conductive film for green light OLED devices, Carbon **172**, 379–389 (2021). <https://doi.org/10.1016/j.carbon.2020.10.027>

[S9] Q. Chen, Y. Ding, M. Xu, Z. Liu, Z. Liu et al., Fast synthesis of silver nanowires at room temperature via ultrasonication-mediated galvanic replacement for flexible transparent electrodes, J. Mater. Chem. C **12**, 1492–1499 (2024). <https://doi.org/10.1039/D3TC03670G>

[S10] W. Li, Y.-Q. Li, Y. Shen, Y.-X. Zhang, T.-Y. Jin et al., Releasing the trapped light for efficient silver nanowires-based white flexible organic light-emitting diodes, Adv. Opt. Mater. **7**, 1900985 (2019). <https://doi.org/10.1002/adom.201900985>

[S11] N. Kim, J. Hwang, H.J. Lee, N.Y. Kwon, J.Y. Park et al., Work-function-tunable metal-oxide mesh electrode and novel soluble bipolar host for high-performance solution-processed flexible TADF-OLED, Nano Energy **105**, 108028 (2023). <https://doi.org/10.1016/j.nanoen.2022.108028>

[S12] E.-H. Ko, H.-K. Kim, Highly transparent vanadium oxide-graded indium zinc oxide electrodes for flexible organic solar cells, Thin Solid Films **601**, 2–6 (2016). <https://doi.org/10.1016/j.tsf.2015.11.067>

[S13] Y. Kim, Y.J. Tak, S.P. Park, H.J. Kim, H.J. Kim, Structural engineering of metal-mesh structure applicable for transparent electrodes fabricated by self-formable cracked template, Nanomater. **7**, 214 (2017). <https://doi.org/10.3390/nano7080214>

[S14] M. Zarei, M. Li, E.E. Medvedeva, S. Sharma, J. Kim et al., Flexible embedded metal meshes by sputter-free crack lithography for transparent electrodes and electromagnetic interference shielding, ACS Appl. Mater. Interfaces **16**, 6382–6393 (2024). <https://doi.org/10.1021/acsami.3c16405>

[S15] P. Liu, B. Huang, L. Peng, L. Liu, Q. Gao et al., A crack templated copper network film as a transparent conductive film and its application in organic light-emitting diode, Sci. Rep. **12**, 20494 (2022). <https://doi.org/10.1038/s41598-022-24672-x>

[S16] M. Cui, X. Zhang, Q. Rong, L. Nian, L. Shui et al., High conductivity and transparency metal network fabricated by acrylic colloidal self-cracking template for flexible thermochromic device, Org. Electron. **83**, 105763 (2020). <https://doi.org/10.1016/j.orgel.2020.105763>

[S17] A.S. Voronin, Y.V. Fadeev, I.V. Govorun, I.V. Podshivalov, M.M. Simunin et al., Cu–Ag and Ni–Ag meshes based on cracked template as efficient transparent electromagnetic shielding coating with excellent mechanical performance, J. Mater. Sci. **56**, 14741–14762 (2021). <https://doi.org/10.1007/s10853-021-06206-4>

[S18] S.-H. Oh, J. Shin, I.-T. Hwang, J.-Y. Sohn, S.-I. Na et al., Facile fabrication of conductive graphitic carbon films as transparent electrodes in organic solar cells by ion beam irradiation of polystyrene films and carbonization, Radiat. Phys. Chem. **215**, 111388 (2024). <https://doi.org/10.1016/j.radphyschem.2023.111388>

[S19] K.A. Nirmal, W. Ren, A.C. Khot, D.Y. Kang, T.D. Dongale et al., Flexible memristive organic solar cell using multilayer 2D titanium carbide MXene electrodes, Adv. Sci. **10**, 2300433 (2023). <https://doi.org/10.1002/advs.202300433>

[S20] P. Wang, M. Jian, C. Zhang, M. Wu, X. Ling et al., Highly stable graphene-based flexible hybrid transparent conductive electrodes for organic solar cells, Adv. Mater. Interfaces **9**, 2101442 (2022). <https://doi.org/10.1002/admi.202101442>

[S21] B.-Y. Wang, E.-S. Lee, Y.-J. Oh, H. Wook Kang, A silver nanowire mesh overcoated protection layer with graphene oxide as a transparent electrode for flexible organic solar cells, RSC Adv. **7**, 52914–52922 (2017). <https://doi.org/10.1039/C7RA10889C>

[S22] M.S.A. Kamel, C.T. Stoppiello, M.V. Jacob, Improved transfer-free sustainable graphene electrode using silver nanowires for organic photovoltaics, ACS Appl. Energy Mater. **6**, 11168–11178 (2023). <https://doi.org/10.1021/acsaem.3c02001>

[S23] J.W. Park, A.V. Takaloo, S.H. Kim, K.R. Son, D.Y. Kang et al., Surface-modified ultra-thin indium zinc oxide films with tunable work function for efficient hole transport in flexible indoor organic photovoltaics, J. Power Sources **489**, 229507 (2021). <https://doi.org/10.1016/j.jpowsour.2021.229507>

[S24] X. Feng, L. Wang, Y.Y.S. Huang, Y. Luo, J. Ba et al., Cost-effective fabrication of uniformly aligned silver nanowire microgrid-based transparent electrodes with higher than 99% transmittance, ACS Appl. Mater. Interfaces **14**, 39199–39210 (2022). <https://doi.org/10.1021/acsami.2c09672>

[S25] Z. Wang, M. Wang, B. Jiao, W. Lu, D. Xu et al., Smooth and mechanically robust random metallic mesh electrode modified by thermally transferred PEDOT: PSS for ITO-Free flexible organic light-emitting diodes, Org. Electron. **106**, 106498 (2022). <https://doi.org/10.1016/j.orgel.2022.106498>

[S26] Y. Zhao, X. Zhou, W. Huang, J. Kang, G. He, High-performance copper nanowire electrode for efficient flexible organic light-emitting diode, Org. Electron. **113**, 106690 (2023). <https://doi.org/10.1016/j.orgel.2022.106690>

[S27] A. Rani, W. Ren, H.J. Lee, S.H. Hong, T.G. Kim, Synthesis, properties, and application of ultrathin and flexible tellurium nanorope films: beyond conventional 2D materials, Small **20**, 2300557 (2024). <https://doi.org/10.1002/smll.202300557>

[S28] S.H. Park, C. Shaozheng, H. Cheon, Y.N. Song, T. Kim et al., One-step direct patterning of flexible and transparent InGaTiO/AgPdCu/InGaTiO multi-layer electrodes for flexible organic light-emitting diodes, J. Alloys Compd. **976**, 172972 (2024). <https://doi.org/10.1016/j.jallcom.2023.172972>

[S29] J. Zhu, D. Han, X. Wu, J. Ting, S. Du et al., Highly flexible transparent micromesh electrodes via blade-coated polymer networks for organic light-emitting diodes, ACS Appl. Mater. Interfaces **12**, 31687–31695 (2020). <https://doi.org/10.1021/acsami.0c07299>

[S30] T.H. Park, W. Ren, H.J. Lee, N. Kim, K.R. Son et al., Efficient TADF-based blue OLEDs with 100% stretchability using titanium particle-embedded indium zinc oxide mesh electrodes, NPG Asia Mater. **14**, 1–13 (2022). <https://doi.org/10.1038/s41427-022-00411-6>

[S31] Z. Jiang, W. Zhang, J. Bao, H. Cheng, X. Zhang et al., The effect of electrodes on microstructures and switching behaviors of ZnO-based resistive memory, Ceram. Int. **46**, 24838–24843 (2020). <https://doi.org/10.1016/j.ceramint.2020.06.267>

[S32] V.-Q. Le, T.-H. Do, J.R.D. Retamal, P.-W. Shao, Y.-H. Lai et al., Van der Waals heteroepitaxial AZO/NiO/AZO/muscovite (ANA/muscovite) transparent flexible memristor, Nano Energy **56**, 322–329 (2019). <https://doi.org/10.1016/j.nanoen.2018.10.042>

[S33] W. Duan, All inorganic and transparent ITO/boehmite/ITO structure by one-step synthesis method for flexible memristor, Solid-State Electron. **186**, 108180 (2021). <https://doi.org/10.1016/j.sse.2021.108180>

[S34] F.M. Simanjuntak, T. Ohno, S. Samukawa, Influence of rf sputter power on ZnO film characteristics for transparent memristor devices, AIP Adv. **9**, 105216 (2019). <https://doi.org/10.1063/1.5125665>

[S35] F.M. Simanjuntak, S. Chandrasekaran, C.-C. Lin, T.-Y. Tseng, ZnO_2_/ZnO bilayer switching film for making fully transparent analog memristor devices, APL Mater. **7**, 051108 (2019). <https://doi.org/10.1063/1.5092991>
